# Erratum

**DOI:** 10.1590/2175-8239-JBN-2018-00030003er

**Published:** 2018-11-14

In the article *"Reflections on End-of-Life Dialysis"* with DOI code number http://dx.doi.org/10.1590/2175-8239-JBN-2018-00030003 published at Brazilian Journal of Nephrology in 2018:

Where it was written:

*In this issue, the author of "Reflections on Endof-Life Dialysis" covered issues seldom addressed in nephrology services: decision-making; shared decision-making; the tools we have; the vulnerability of physicians; healthcare worker and aregiver burnout.*

Should read:

*In this issue, the author of "Reflections on Endof-Life Dialysis" covered issues seldom addressed in nephrology services: decision-making; shared decision-making; the tools we have; the vulnerability of physicians; healthcare worker and aregiver burnout.^8^*

Where it was written:

*this decision is the patient's mental state^8^.*

Should read:

*this decision is the patient's mental state^9^.*

Where it was written:

*tools that might be used in patient assessment.^9^*

Should read:

*tools that might be used in patient assessment.^10^*

Where it was written:

*scores for clinical management and shared decision support^10^.*

Should read:

*scores for clinical management and shared decision support^11^.*

Where it was written:

*The BANSAL score^10^ is useful for predicting the (...)*

Should read:

*The BANSAL score^11^ is useful for predicting the (...)*

Where it was written:

*The REIN^10^ (Renal Epidemiology and Information Network) (...)*

Should read:

*The REIN^11^ (Renal Epidemiology and Information Network) (...)*

Where it was written:

*the choice between modes of dialysis and conservative management.^10^*

Should read:

*the choice between modes of dialysis and conservative management.^11^*

Where it was written:

7. Carson RC, Juszczak M, Davenport A, Burns A. Is maximum conservative management an equivalent treatment option to dialysis for elderly patients with significant comorbid disease? Clin J Am Soc Nephrol 2009;4:1611-9.

8. Kee F, Patterson CC, Wilson EA, McConnell JM, Wheeler SM, Watson JD. Stewardship or clinical freedom? Variations in dialysis decision making. Nephrol Dial Transplant 2000;15:1647-57.

9. Antonelli Incalzi R, Aucella F, Leosco D, Brunori G, Dalmartello M, Paolisso G. Assessing Nephrological Competence among Geriatricians: A Proof of Concept Internet Survey. PLoS One 2015;10:e0141388. DOI: 10.1371/journal. pone.0141388.

10. Farrington K, Covic A, Aucella F, Clyne N, de Vos L, Findlay A; ERBP Guideline Development Group. Clinical Practice Guideline on management of older patients with chronic kidney disease stage 3b or higher (eGFR<45 mL/min/1.73 m2). Nephrol Dial Transplant 2016;31:ii1-ii66. Erratum in Nephrol Dial Transplant 2017;32:740-1.

Should read:

7. Carson RC, Juszczak M, Davenport A, Burns A. Is maximum conservative management an equivalent treatment option to dialysis for elderly patients with significant comorbid disease? Clin J Am Soc Nephrol 2009;4:1611-9.

8. Castro MCM. Reflections on end-of-life dialysis. J Bras Nefrol 2018; 40:232-40

9. Kee F, Patterson CC, Wilson EA, McConnell JM, Wheeler SM, Watson JD. Stewardship or clinical freedom? Variations in dialysis decision making. Nephrol Dial Transplant 2000;15:1647-57.

10. Antonelli Incalzi R, Aucella F, Leosco D, Brunori G, Dalmartello M, Paolisso G. Assessing Nephrological Competence among Geriatricians: A Proof of Concept Internet Survey. PLoS One 2015;10:e0141388. DOI: 10.1371/journal. pone.0141388.

11. Farrington K, Covic A, Aucella F, Clyne N, de Vos L, Findlay A; ERBP Guideline Development Group. Clinical Practice Guideline on management of older patients with chronic kidney disease stage 3b or higher (eGFR<45 mL/min/1.73 m2). Nephrol Dial Transplant 2016;31:ii1-ii66. Erratum in Nephrol Dial Transplant 2017;32:740-1.

In the article *"IgA nephropathy in Salvador, Brazil: a more aggressive disease?"* with DOI code number http://dx.doi.org/10.1590/2175-8239-JBN-2018-00030002 published at Brazilian Journal of Nephrology in 2018:

Where it was written:

*patterns of 32 patients with IgAN in Salvador, Brazil.*

Should read:

*patterns of 32 patients with IgAN in Salvador, Brazil.^7^*

Where it was written:

*analyses according to the Oxford classification of IgAN.^7 8^*

Should read:

*analyses according to the Oxford classification of IgAN.^8,9^*

Where it was written:

6. Soares MF, Caldas ML, Dos-Santos WL, Sementelli A, Furtado P, Araujo S, et al. IgA nephropathy in Brazil: apropos of 600 cases. Springerplus 2015;4:547.

7. Kang SH, Choi SR, Park HS, Lee JY, Sun IO, Hwang HS, et al. The Oxford classification as a predictor of prognosis in patients with IgA nephropathy. Nephrol Dial Transplant 2012;27:252-8.

8. Barbour SJ, Espino-Hernandez G, Reich HN, Coppo R, Roberts IS, Feehally J, et al; Oxford Derivation, North American Validation and VALIGA Consortia. The MEST score provides earlier risk prediction in IgA nephropathy. Kidney Int 2016;89:167-75.

Should read:

6. Soares MF, Caldas ML, Dos-Santos WL, Sementelli A, Furtado P, Araujo S, et al. IgA nephropathy in Brazil: apropos of 600 cases. Springerplus 2015;4:547.

7. Souza BN, Tavares MB, Soares MFS, Santos WLC. IgA Nephropathy in Salvador, Brazil. Clinical and laboratory presentation at diagnosis. J Bras Nefrol 2018;40:241-6

8. Kang SH, Choi SR, Park HS, Lee JY, Sun IO, Hwang HS, et al. The Oxford classification as a predictor of prognosis in patients with IgA nephropathy. Nephrol Dial Transplant 2012;27:252-8.

9. Barbour SJ, Espino-Hernandez G, Reich HN, Coppo R, Roberts IS, Feehally J, et al; Oxford Derivation, North American Validation and VALIGA Consortia. The MEST score provides earlier risk prediction in IgA nephropathy. Kidney Int 2016;89:167-75.

In the article *"The intricate relationship between gut and kidney"* with DOI code number http://dx.doi.org/10.1590/1678-4685-JBN-2018-00020001 published at Brazilian Journal of Nephrology in 2018:

Where it was written:

*Based on the study conducted by Andrade et al. and in the literature, it seems reasonable to suggest that uremia affects the intestinal epithelium by inducing the inflammatory process and compromising the intercellular junctions. However (...)*

Should read:

*Based on the study conducted by Andrade et al. and in the literature, it seems reasonable to suggest that uremia affects the intestinal epithelium by inducing the inflammatory process and compromising the intercellular junctions.^8^ However (...)*

Where it was written:

7. Andrade LS, Ramos CI, Cuppari L. The cross-talk between the kidney and the gut: implications for chronic kidney disease. Nutrire 2017;42:27. DOI: 10.1186/s41110-017-0054-x

Should read:

7. Andrade LS, Ramos CI, Cuppari L. The cross-talk between the kidney and the gut: implications for chronic kidney disease. Nutrire 2017;42:27. DOI: 10.1186/s41110-017-0054-x

8. Andrade LS, Dalboni MA, Carvalho JTG, Grabulosa CC, Pereira NBF, Aoike DT, Cuppari L. In vitro effect of uremic serum on barrier function and inflammation in human colonocytes. J Bras Nefrol 2018;40:2016-23.

